# The Interaction of Viruses with the Cellular Senescence Response

**DOI:** 10.3390/biology9120455

**Published:** 2020-12-09

**Authors:** Rocío Seoane, Santiago Vidal, Yanis Hichem Bouzaher, Ahmed El Motiam, Carmen Rivas

**Affiliations:** 1 Centro de Investigación en Medicina Molecular y Enfermedades Crónicas (CIMUS), Universidad de Santiago de Compostela, 15706 Santiago de Compostela, Spain; rocioseoane.abelenda@usc.es (R.S.); santiago.vidal@usc.es (S.V.); yanishichem.bouzaher@rai.usc.es (Y.H.B.); ahmed.hassanahmedmohammed@usc.es (A.E.M.); 2 Centro Nacional de Biotecnología (CNB), CSIC, 28049 Madrid, Spain

**Keywords:** senescence, senolytics, virosenolytics, virus

## Abstract

**Simple Summary:**

Cellular senescence is considered a stress response that protects cells against malignant transformation, facilitates tissue repair and development, and prevents virus replication. However, excessive accumulation of senescent cells is associated with chronic diseases such as age-related disorders, cancer, inflammatory diseases and virus replication. The relationship between virus and cellular senescence is proving to be very complex. Cellular senescence can be induced in response to virus infection restricting virus propagation. Some viruses are able to exploit the senescence program to improve their replication, while others have developed strategies to subvert senescence. Therapeutic approaches to eliminate senescent cells may be used as a mechanism to ameliorate age-related diseases, but they may have an impact on virus replication. Here we review the available evidence revealing an interplay between cellular senescence and virus replication. We also discuss the consequences that treatment with senolytic agents may have on virus replication.

**Abstract:**

Cellular senescence is viewed as a mechanism to prevent malignant transformation, but when it is chronic, as occurs in age-related diseases, it may have adverse effects on cancer. Therefore, targeting senescent cells is a novel therapeutic strategy against senescence-associated diseases. In addition to its role in cancer protection, cellular senescence is also considered a mechanism to control virus replication. Both interferon treatment and some viral infections can trigger cellular senescence as a way to restrict virus replication. However, activation of the cellular senescence program is linked to the alteration of different pathways, which can be exploited by some viruses to improve their replication. It is, therefore, important to understand the potential impact of senolytic agents on viral propagation. Here we focus on the relationship between virus and cellular senescence and the reported effects of senolytic compounds on virus replication.

## 1. Introduction

Viral infection triggers the activation of many cellular stress–response pathways, such as heat shock, oxidative-stress or DNA damage response (DDR), that can result in the induction of apoptosis or autophagy. In most cases, these cellular responses contribute to controlling virus replication. However, some viruses have developed strategies to avoid these antiviral responses or subvert them for their own benefit [1,2]. Another consequence of the activation of stress response pathways by viruses may be the induction of senescence. For some viruses, the unspecific induction of senescence is part of the antiviral response, limiting virus replication. Reinforcing the hypothesis that senescence contributes to control virus replication, some viruses encode for proteins that specifically inhibit this process. In contrast, some viruses are able to usurp the senescence pathway to promote virus production. For example, some of the transcriptionally upregulated genes in senescent cells are cellular proteins that work as viral receptors. Moreover, the characteristic stable cell cycle arrest that defines cell senescence can benefit some viral agents. For example, the human immunodeficiency virus (HIV) is more transcriptionally active in G2 [3], and a G2 cell cycle arrest increases the number of integrated HIV provirus [4]. In addition, a G2 arrest can also modulate viral or cellular genes that are important for the completion of the virus life cycle, as proposed for the enhanced expression of papillomavirus capsid proteins of human papillomavirus type 6 (HPV6) and bovine papillomavirus type 1 (BPV1) [5]. Similar to what occurs with other antiviral responses, the outcome of senescence depends on the virus.

Cellular senescence denotes a condition of stable cell cycle arrest in which senescent cells do not replicate but stay viable and metabolically active. This process can be beneficial and protect against cancer or other types of stress, but an excess of senescent cells can favor cancer progression [6]. Senescent cells are characterized by metabolic and morphological alterations, reorganization of the chromatin, altered gene expression and the secretion of a variety of cytokines, growth factors, proteases, and chemokines called senescent-associated secretory phenotype (SASP) [7,8].

Although many biomarkers of senescence have been identified, most of them lack specificity or are restricted to particular conditions. Among the diverse hallmarks of cellular senescence, some of the most characteristic ones are a persistent DDR; a stable cell cycle arrest mediated by the p53/p21^Cip1^ and p16^INK4a^/pRb pathways; morphological changes characterized by enlarged size, flattened shape, extensive vacuolization and multinucleation; upregulation of the cell cycle regulators p16^INK4a^ and p21^Cip1^; formation of senescence-associated heterochromatin foci (SAHF); the acquisition of a senescence-associated secretory phenotype (SASP); and increased levels of beta-galactosidase (SA-beta-gal) activity at pH 6.0 [9].

Cellular senescence can occur in response to different stimuli, including chemotherapeutic treatment, oxidative or genotoxic stress, ionizing radiation, telomere shortening, oncogenic signaling or virus infection [10]. DNA damage is a key event for the induction of cell senescence in response to many of these stimuli, such as treatment with chemotherapeutic drugs, oxidative stress, the shortening of telomeres, or oncogenic stress [11].

A factor linked to both DNA damage and senescence is interferon (IFN) signaling. Persistent beta IFN treatment triggers a DNA damage signaling pathway and senescence [12], and DNA damage induces type I IFN and stimulate IFN signaling, which amplifies DNA damage responses and promotes a p53-dependent senescence program [13,14,15].

In this review, we focus on the interplay between viruses and senescence and the potential of senolytic drugs as antivirals.

## 2. Virus and Senescence

### 2.1. Virus and Senescence-Associated Pathologies 

Cellular senescence is considered a mechanism to prevent malignant transformation of damaged cells, to facilitate tissue repair and development, and to control virus replication. However, senescence can also contribute to the development of different pathologies such as age-related disorders, cancer, inflammatory diseases and virus replication [5,16]. Although the upregulation of senescence-associated pathways is related to some pathological conditions that can be triggered by a viral infection, the existence of a direct relationship between virus infection and senescence induction in most of these diseases has not been proved.

One of the few pathologies clearly related to both senescence and virus infection is HIV-related osteoporosis and osteopenia. The maintenance of bone remodeling requires an adequate balance between osteoblast generation-osteoclast resorption and an alteration of this balance leads to osteoporosis or osteopenia [17]. Cellular senescence is considered one of the key factors in this disease, and it is clearly established that HIV infection is a risk factor for this pathological condition [18,19,20]. The secretion of two HIV proteins, Tat and Nef, to the media promotes human bone marrow mesenchymal stem cell senescence through induction of inflammation and reduction of autophagy, altering osteoblastic differentiation and inducing osteopenia or osteoporosis [21].

### 2.2. Cellular Senescence in Response to Virus Infection

The fact that many viruses activate the cellular DDR [22] and that virus infection induce the production of IFN suggests that viruses may be a stimulus for senescence induction. The induction of senescence in response to virus infection can result from the activation of different pathways (Table 1). Infection of immortalized epithelial cells as well as of the respiratory epithelia of mice with the human respiratory syncytial virus (HRSV) induces double-stranded breaks (DSB) caused by the generation of reactive oxygen species (ROS) at the mitochondria leading to activation of a DDR and the induction of senescence. A contribution of the HRSV-induced senescence to the physiopathology of the infection has been proposed since the senescence state, and DNA damage induction still persists in the lungs of the infected mice [23]. Similarly, infection with Merkel cell polyomavirus (MCPyV) also activates a host DDR resulting in the induction of senescence of normal human dermal fibroblasts [20]. Expression of the large T antigen of MCPyV has been demonstrated to induce DSB, the activation of ataxia–telangiectasia mutated (ATM) protein kinase and the phosphorylation of p53 and KRAB-associated protein 1 (KAP-1). The accumulation of phosphorylated KAP-1 has been associated with a G2 phase arrest, a mechanism that may avoid viral DNA replication [24,25]. 

Infection of the human umbilical vein endothelial cells (HUVEC) with dengue virus (DENV) also leads to the expression of SA-beta-gal, cell cycle arrest and morphologic changes typical of senescent cells [26], and this process has been proposed to contribute to the pathogenesis of the virus. However, whether the DENV-induced senescence occurs through a DNA damage-mediated pathway is unknown.

Another mechanism that mediates virus-induced cellular senescence is cell-to-cell fusion, a strategy that may facilitate viral spreading, avoiding detection by the immune system [27]. Infection of normal human lung fibroblast (IMR90) cells with measles virus (MV) triggers cellular senescence as revealed by the reduction of cell proliferation, the SA-beta-gal staining, the increase in the expression of p53 and p21 or the induction in the expression of SASP components such as interleukin-8 (IL-8) or the C–C motif chemokine ligand 5 (CCL5). The authors also reported an increase in DNA damage and p53 phosphorylation in IMR90 cells expressing the MV protein ERWVE, which facilitates the maintenance of the senescence state of the cells. Induction of senescence by MV was not restricted to IMR90. MV infection also triggered senescence in the adenocarcinoma cell line A549, and this induction occurs in a p53-dependent manner [28]. The authors propose that this process may facilitate the recognition and elimination of the infected cells by the immune system.

Another virus that has been proposed to trigger senescence through activation of p53, as well as the p16^INK4a^ pathways, is the human cytomegalovirus (HCMV). Infection of fibroblasts with HCMV induces cell cycle arrest and premature senescence, probably mediated by the expression of the viral IE2 protein [29,30]. Similar to HCMV IE2 protein, the expression of other individual viral proteins can also promote cellular senescence. This is the case of the HIV Tat and Nef proteins, which can induce senescence of human bone marrow mesenchymal stem cells [21], as mentioned above. Inhibition of autophagy seems to mediate the HIV Nef-induced senescence while Tat triggers senescence through the nuclear factor kappa B (NF-kB) pathway [21,31]. Moreover, HIV Tat may also trigger senescence by additional mechanisms. Thus, expression of HIV Tat in endothelial cells or in transgenic mice upregulates the expression of the microRNA miR-34a [32], a molecule that targets sirtuin 1 (SIRT1), leading to the induction of senescence [33,34]. Together with Tat and Nef, other HIV proteins can also increase the expression of this senescence-associated microRNA and contribute to the induction of cell senescence, as revealed after the expression of the gp120 protein from X4 and R5 HIV-1 strains in endothelial cells [35]. Analysis of senescence markers such as p16^INK4a^, p53 or SA-beta-gal activity revealed that expression of Tat and Nef proteins from the simian immunodeficiency virus (SIV) in adipose tissue and human adipose stem cells also results in the induction of senescence [36]. Activation of the senescence program by both proteins occurs through an oxidative stress pathway. The pro-senescent activity of HIV proteins has been proposed to contribute to the pathology in infected people, such as in cardiovascular diseases. 

Senescence induction also results from infection with other tumor viruses such as hepatitis C virus (HCV) or hepatitis B virus (HBV). Senescent hepatocytes were found in chronic hepatitis due to HCV [37]. In addition, a correlation between liver fibrosis observed in chronic HCV infections and cellular senescence has been reported. Cellular senescence in liver cells infected with HCV has been suggested to occur as a consequence of the telomere shortening produced in response to the oxidative stress caused in the mitochondria by the HCV core protein [37,38]. Analysis of liver tissue from patients with chronic HBV infection also revealed an association between HBV infection and senescence-associated markers [39].

Interestingly, the NS1 protein of some influenza A virus (IAV) strains can also trigger cellular senescence. Thus, the increase in nitric oxide synthase expression and nitric oxide release by neuro2a cells and primary cultured mouse cortical neurons in response to NS1 protein from H7N9 IAV expression can activate a cellular senescence program [40].

### 2.3. Cell Senescence as an Antiviral Response

Cellular senescence can contribute to dysfunctional viral-sensing mechanisms and thus affect virus replication. Senescence of primary or tumor cells, independently of the stimuli involved in its induction, is a mechanism to control vesicular stomatitis virus (VSV) infection [44]. Importantly, the control of VSV propagation by senescence was also demonstrated in mice. Senescence induction in a mouse model of bleomycin-induced lung injury reduced the VSV recovery from the lungs of the infected mice [44].

Senescence of HUVEC cells was also reported to inhibit DENV infection [26]. However, this effect seems to be cell type-dependent since senescence induction in monocytes has been shown to trigger an increase in the expression of the dendritic cell-specific intercellular adhesion molecule-3 (ICAM-3) grabbing nonintegrin (DC-SIGN) receptor facilitating DENV infection [45].

Finally, a negative role of senescence on MCPyV infection has been reported. Siebels et al. (2020) demonstrated that KAP-1 is a restriction factor for MCPyV infection and that replication of the virus induces the phosphorylation of KAP-1 and the subsequent cellular senescence [25]. Therefore, it has been proposed that senescence is a host defense mechanism against MCPyV [25].

### 2.4. Viral Proteins That Inhibit Cellular Senescence

Cellular senescence may contribute to the antiviral activity of IFN [46]. To replicate in the host, viruses require mechanisms to subvert this antiviral response. Therefore, some viruses have developed proteins able to counteract cell senescence (Table 2).

The deregulation of cell proliferation that occurs after the initial infection of primary cells in response to the expression of latent viral oncoproteins from the Epstein–Barr virus (EBV) or Kaposi’s sarcoma-associated herpesvirus (KSHV) elicits the activation of a DNA damage response [47,48] and the induction of oncogene-induced senescence [41,42]. Early after B-cell infection with EBV, the expression of the latent proteins EBNA-LP and EBNA2 induces the transcriptional transactivation of cellular genes that control entry into the cell cycle and the activation of an ATM/Chk2-dependent DDR [47]. Activation of the ATM kinase is also the mechanism inducing the DDR signaling in response to KSHV infection [48]. However, the expression of the EBV latent proteins EBNA3C and LPM1 of EBV has been shown to attenuate the DNA damage response [47] and to block the p16^INK4a^-Rb pathway promoting senescence bypass [49,50]. Similarly, expression of the KSHV latent proteins vCyclin and v-FLIP also inhibits senescence. KSHV vCyclin forms active kinase complexes with Cdk6 that are resistant to the inhibition by p21^Cip1^, p16^INK4a^ and p27^Kip1^ [51] and induces p27 degradation [52] suppressing replicative senescence in primary human lymphatic endothelial cells [53] as well as senescence triggered by NF-kB hyperactivation [54]. Suppression of autophagy has been proposed as the mechanism that mediates the senescence bypass by KSHV v-FLIP [55].

The HBx protein of HBV has a crucial role in the pathogenesis of hepatocellular carcinoma (HCC). Expression of the C-terminal truncated HBx protein has been reported to induce senescence through the induction of p16^INK4a^ and p21^Cip1^ expression and the downmodulation of pRb phosphorylation [43]. However, the expression of the full-length HBx protein diminishes the expression of DNA methyltransferases, downmodulating the expression of p16^INK4^ and p21^Cip1^ and overcoming senescence [56,57].

Other two viral proteins able to inhibit senescence are the human papillomavirus (HPV) E6 and E7 proteins. Among the different functions attributed to HPV E6 and E7 proteins is the binding of HPV E7 to the active form of the retinoblastoma family of tumor suppressor proteins inducing its destabilization, the degradation of p53 or the activation of telomerase [58]. Although co-expression of both proteins bypasses replicative senescence in keratinocytes, only the inhibition of pRb and the stimulation of telomerase activity was essential for this inhibition [59].

### 2.5. Hijacking Cellular Senescence by Virus

Some viruses can exploit the senescence program to increase their replication rate through a variety of mechanisms. One example is viruses that benefit from the increase in viral receptors that occurs when cells go to senescence, such as DENV. DENV can attach to DC-SIGN to infect cells [60], and this receptor is upregulated in senescent cells, which facilitates its infection by DENV [45,60]. The exploitation of the senescence program by DENV may be limited by the cell type. Thus, it has been reported that senescence has a negative impact on the infectivity of HUVEC cells by DENV [26]. It has been speculated that increased DC-SIGN expression in senescent monocytes might increase infectivity by other viruses, including IAV, HIV, Ebola, HCV, cytomegalovirus and severe acute respiratory syndrome coronavirus (SARS-CoV) since these pathogens can be captured by DC-SIGN [61].

A reduction in the virus-induced type I IFN expression in senescent cells is the mechanism proposed by Kim et al. (2016) to explain the increased susceptibility of primary human bronchial epithelial cells and human dermal fibroblasts undergoing replicative senescence to both IAV and varicella-zoster virus (VZV) relative to non-senescent cells [62]. Higher susceptibility of senescent cells to VZV has been proposed as the cause of the relatively high incidence of zoster in aging patients together with immunosenescence.

The increased susceptibility of senescent cells to some viral infections makes senescence a potential target to control viral replication.

### 2.6. Utility or Risks of Using Senolytic Drugs to Treat Virus Infection

The relevance of senescence in some pathogenic conditions has led to the development of senolytics, compounds able to selectively eliminate senescent cells [63], as potential treatments for several diseases [64,65,66]. Since senescence may alter (upregulate or downmodulate) the replication capacity of different viruses, it is important to evaluate the effect of drugs with senolytic activities on virus replication.

Dasatinib is a potent kinase inhibitor whose main targets are the Src family of kinases and probably other tyrosine kinases [67,68]. Its ability to inhibit Src and Bcr-Abl kinases [67,69,70] is the main reason for its utility for the treatment of patients with chronic myeloid leukemia (CML), especially for those resistant to imatinib treatment [71]. Moreover, dasatinib seems to be effective in the selective elimination of senescent cells [68] and also appears to be effective in suppressing renal fibrosis and improve renal function in several animal models [72]. In addition, its combination with other senolytic agents revealed improved physical function in patients with idiopathic pulmonary fibrosis [73]. Treatment with dasatinib has an impact on the replication of different viruses such as the flavivirus DENV and HCV. Treatment of DENV infected cells with dasatinib inhibits DENV replication, particle assembly and secretion, likely through the inhibition of the Src-family kinases [74,75]. Inhibition of the Abl kinase has been proposed as the mechanism through which dasatinib blocks the entry of HCV [76,77]. Several alphaviruses have also been reported to be sensitive to the inhibition of Src family kinases. Thus, treatment of cells infected with chikungunya virus, Mayaro virus, o’nyong-nyong virus, Ross river virus or Venezuelan equine encephalitis virus with dasatinib decreases the translation efficiency of viral RNAs, limiting their replication [78]. Src protein tyrosine kinases play a relevant role at various stages of HIV-1 entry, and its inhibition restricts HIV-1 entry in activated primary CD4 + T cells [79] and preserves the antiviral activity of the sterile alpha motif and histidine-aspartic domain-containing protein 1 (SAMDH1) [80,81]. Other viruses, such as poliovirus, are not affected by this drug [74]. In contrast, there are numerous reports describing the reactivation of latent viral infections in CML patients treated with Src tyrosine kinase inhibitors, including dasatinib or imatinib, likely due to the downmodulation of the T-cell mediated immune response caused by these drugs. Thus, CML patients treated with these drugs present more predisposition for infection with or reactivation of different viruses such as cytomegalovirus, human herpesvirus 6, VZV or SARS-CoV-2 [82,83,84,85,86,87]. Additionally, although dasatinib treatment has been reported to inhibit the replication of HBV [88], enhanced HBV replication and reactivation has been reported in CML patients [89,90].

Navitoclax (also known as ABT-263) belongs to a class of senolytic agents whose mechanism of action is the inhibition of the Bcl-2 family of anti-apoptotic proteins [91,92]. It has been validated in different preclinical models demonstrating its capacity to kill senescent cells, although with high toxicity [93]. Evaluation of navitoclax on virus replication revealed that it induces premature apoptosis of cells infected with IAV leading to the attenuation of the production of proinflammatory and antiviral cytokines [94]. Importantly, treatment with navitoclax of mice infected with IAV led to an imbalance in cytokine production that, together with the inability of the immune system to clear the virus, reduced the survival rate of IAV-infected mice. Although the authors demonstrated that navitoclax treatment also induced the premature apoptosis of cells infected with other viruses such as herpes simplex virus type-1 (HSV-1), MV, vaccinia virus (VACV), herpes simplex virus type-2 (HSV-2), influenza B virus (IBV), Bunyanwera virus (BUNV) or Sindbis virus (SINV) in vitro, they did not evaluate whether this increased apoptosis accelerated or attenuated virus infection in vitro or in vivo. A reduction in virus replication resulting from premature and specific death of the cells infected with IAV, IBV, Middle East respiratory syndrome (MERS-CoV), Zika virus (ZIKV), HBV, HSV-1, HSV-2 and echovirus 1 and 6 upon navitoclax treatment has been recently reported [95]. The selective killing of HIV-infected primary cells during productive infection by navitoclax has been proposed as a strategy of eliminating host cells capable of producing HIV [96].

HSP90 inhibitors such as geldanamycin, 17-DMAG (alvespimycin), and 17-AAG (tanespimycin) constitute other classes of agents with senolytic activity in mouse and human cells. These compounds destabilize the phosphorylated form of protein kinase B (PKB/AKT), resulting in apoptosis of senescent cells [97]. Numerous articles demonstrate that HSP90 is required for most viral protein homeostasis. Therefore these drugs can exert antiviral activity against a wide variety of viruses. Inhibition of HSP90 through treatment with geldanamycin reduced the replication of VSV, paramyxovirus SV5, human parainfluenza virus type-2 (HPIV-2), human parainfluenza virus type-3 (HPIV-3), Simian virus 41 (SV41), La Crosse bunyavirus, Zaire Ebola virus, VACV, HCV, HSV-2, and poliovirus [98,99,100,101,102,103].

Another frequently used senolytic agent is quercetin [68]. Quercetin belongs to a group of natural compounds found in a wide variety of plants and fruits. The senolytic activity of quercetin is mediated by the modulation of different signaling pathways and gene expressions such as NF-kB, cyclin D1, Bax, Bcl-2, PARP and Gadd45 [104]. Recently, these compounds acquired high interest because different screenings and preliminary studies propose quercetin as a potential drug against SARS-CoV-2 [105,106,107,108] and ZIKV replication [109]. Quercetin has been proved to inhibit ZIKV replication by binding to ZIKV NS2B-NS3 protease and inhibiting its catalytic activity [110,111]. In addition, quercetin also prevents ZIKV to enter into the host cells [109], likely through a direct action on the viral particle, as described for other flavonoid agents like epigallocatechin gallate (EGCC) [112]. It has been reported that this compound binds to the surface of the HIV envelope and destabilizes the viral particle [113]. A similar interaction with and inhibition of the DENV NS2B-NS3 protease has also been reported [114,115,116]. In addition, quercetin has also been demonstrated to exert antiviral activity against other viruses. Quercetin inhibits HCV by many different mechanisms directed either against the host or the virus. Thus, it affects virion integrity, decreases internal ribosome entry site (IRES) activities [117], inhibits NS5A-driven IRES-mediated translation of the viral genome [118,119], and inhibits HCV replication [120]. Moreover, quercetin also downmodulates the synthesis of triacylglycerol (TAG), partially through modulating diacylglycerol acyltransferase (DGAT) activity, affecting the trafficking of the HCV core protein to lipid droplets (LDs), an essential process for infectious virion production [121]. This drug has also been shown to be effective against IAV. Quercetin interacts with the hemagglutinin of different IAV strains, blocking their entry into the cell [122]. Herpesviruses are also blocked by this drug. In this case, quercetin downmodulates the expression of immediate-early genes of HCMV and VZV [123], whereas several activities seem to mediate its effect on HSV-1. Quercetin inhibits binding and entry of HSV-1 in host cells and downmodulates NF-kB activation inhibiting viral gene expression [124]. Inhibition of the expression of TLR-3 has been proposed as the mechanism by which quercetin inhibits NF-kB and IRF3 [125].

A novel class of broad-spectrum senolytics is cardiac glycosides such as ouabain or digoxin [126,127]. This family of compounds targets the Na+/K+ATPase pump causing an imbalanced electrochemical gradient within the cell leading to depolarization and acidification. Cardiac glycosides have been demonstrated to have a broad antiviral activity such as anti- alphavirus [128], cytomegalovirus [129,130,131], herpesvirus [132,133], or HIV [134,135], among others, and the mechanism of action depends on the viral agent. Ouabain downmodulates the expression of both viral RNA and antigens in cells infected with Sendai virus, likely by preventing the intracellular accumulation of K ion required for the exponential growth of the virus [136]. Ouabain and digoxin also inhibit SARS-CoV-2 replication [137] as well as the cellular entry of VSV, mouse hepatitis virus (MHP), feline infectious peritonitis virus (FIPV) and MERS-CoV through a mechanism mediated by Src [138]. In this study, the authors did not find any effect of these compounds on IAV. However, more recent studies have found that cardiac glycosides decrease IAV replication in alveolar epithelial cells by decreasing intracellular potassium, which triggers the inhibition of protein translation [139].

The ability of some viruses to exploit the senescence pathway to improve their replication opens a window of opportunities to use those viruses as potential senolytic agents. Thus, infection with MV has been reported to accelerate the lysis of a panel of cancer cell lines induced into senescence after treatment with chemotherapeutic drugs [140]. The combinatorial effect of measles-based virotherapy, together with the chemotherapeutic agent that induces senescence, gemcitabine, on human pancreatic cancer cell lines, has also been evaluated [141]. The authors found that the combination was significantly more effective than the individual treatments reducing cell viability. The use of MV as a senolytic agent is possible due to the ability of the virus to replicate in senescent cells and induce their lysis even faster than in non-senescent cells. An increase in the cellular receptors intercellular adhesion molecule-1 (ICAM-1) and decay-accelerating factor (DAF) in response to doxorubicin and the consequently improved infection by the oncolytic Coxsackievirus A21 may explain why the combination of this viral agent with the senescence-inducing agent doxorubicin also resulted in greater tumor reduction compared to either agent alone [142]. Another oncolytic virus that replicates more efficiently in senescent than in normal cells is IAV. Therefore, an IAV adapted to avoid pathogenicity may be a potentially useful “virosenolytic” agent.

## 3. Conclusions

**Figure 1:**
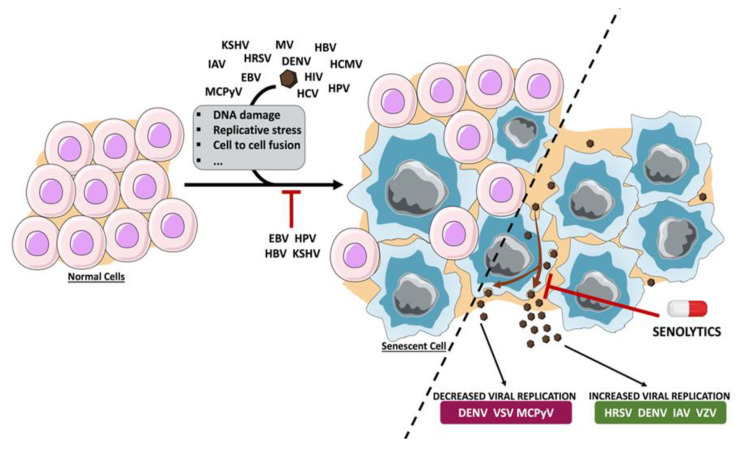
Schematic representation of the induction and/or inhibition of senescence by viruses and the effect of senescence on virus replication. Upon virus infection, cells can enter into senescence as a consequence of DNA damage, replicative stress or cell-to-cell fusion, among others. Cellular senescence limits the replication of different viral agents, whereas it contributes to the efficient replication of others. Consequently, targeting the senescent cells using senolytic agents has a negative or positive effect on virus infection, depending on the virus. Dengue virus (DENV), Epstein–Barr virus (EBV), hepatitis B virus (HBV), human cytomegalovirus (HCMV), human immunodeficiency virus (HIV), human papillomavirus (HPV), human respiratory syncytial virus (HRSV), influenza A virus (IAV), Kaposi’s sarcoma-associated herpesvirus (KSHV), Merkel cell polyomavirus (MCPyV), measles virus (MV), varicella-zoster virus (VZV), vesicular stomatitis virus (VSV).

Prolonged IFN treatment or infection with some viruses can promote cellular senescence, which can protect against the infection with these or other viral agents, but it may contribute to the physiopathology of the infection. Some viral agents have developed strategies to prevent cellular senescence, thereby promoting virus replication and virus-related diseases. Others have evolved different mechanisms to exploit the senescence program for their own benefit. Senescence-targeted therapies have proved effective in removing senescent cells from animal models and humans and have been proposed as a therapeutic strategy to delay, prevent or treat different age-associated pathologies. Senolytic strategies may also be useful to combat the infection with those viruses that benefit from cellular senescence (Figure 1). One potential problem of using oncolytic viruses as senolytic agents is the antiviral immunity that can decrease the effectiveness of the viral agent. In addition, the proinflammatory response promoted by the virus will be added to the extracellular factors that comprise the SASP and that evoke immune responses. Could this strategy result in excessive inflammation? Finally, it is also important to consider that many clinical data reveal the reactivation of latent viral infections in response to senolytic drugs. What would be the effect of a virus coinfection? More studies are needed to define the benefits and risks of these compounds on virus-related diseases.

## Figures and Tables

**Table 1:** Induction of senescence by viruses.

Virus	Mechanism	References
Human respiratory syncytial virus (HRSV)	Oxidative-stress-mediated DNA damage	[23]
Human cytomegalovirus (HCMV)	The HCMV IE2 protein transcriptionally upregulates p16^INK4a^	[29,30]
Epstein–Barr virus (EBV)	Replicative stress and DNA damage	[41]
Kaposi’s sarcoma-associated herpesvirus (KSHV)	Replicative stress and DNA damage	[42]
Merkel cell polyomavirus (MCPyV)	ATM-dependent DDR	[25]
H7N9 influenza A virus (IAV)	Viral NS1 protein increases nitric oxide synthase expression and nitric oxide release	[40]
Human immunodeficiency virus (HIV)	HIV Tat protein activates NF-kB	[21,31]
	HIV Tat, and gp120 proteins upregulate miR34a	[32,35]
	HIV Nef protein inhibits autophagy	[21]
Hepatitis B virus (HBV)	The C-terminally truncated HBx protein of HBV upregulates p16^INK4a^ and p21^Cip1^ and downmodulates pRb	[39,43]
Hepatitis C virus (HCV)	Telomere shortening	[37,38]
Measles virus (MV)	Cell to cell fusion	[28]
Dengue virus (DENV)	Unknown mechanism	[26]

**Table 2:** Inhibition of senescence by viruses.

Virus	Mechanism	References
Epstein–Barr virus (EBV)	EBV latent proteins EBNA3C and LMP1 attenuate DDR and block p16^INK4a^-pRb pathway	[47,49,50]
Kaposi’s sarcoma-associated herpesvirus (KSHV)	KSHV vCyclin protein forms active kinase complexes with Cdk6 and induces p27 degradation KSHV vFLIP protein suppresses autophagy	[51,52,53,54,55]
Hepatitis B virus (HBV)	HBV HBx protein downmodulates p16^INK4a^ and p21^Cip1^	[56,57]
Human papillomavirus (HPV)	HPV E6 and E7 proteins destabilize pRb and activate telomerase	[58,59]
